# GPR43 regulates mitochondrial apoptosis through the cyclophilin D pathway in Alzheimer’s disease

**DOI:** 10.1186/s10020-025-01269-4

**Published:** 2025-06-04

**Authors:** Xiaoqin Wang, Shijing Wu, Zhangjing Deng, Mengyu Yan, Dandan Wang, Maojun Yang, Fuxing Zhong, Jiaqi Song, Lihua Chen, Yingxi Chen, Qi Tian, Weihua Yu, Yang Lü

**Affiliations:** 1 Department of Geriatrics, The First Affiliated Hospital of Chongqing Medical University, No. 1 You Yi Road, Yu Zhong District, Chongqing, 400016 China; 2 Institutes of Neuroscience, Chongqing Medical University, No.1 Yixuayuan Road, Yu zhong District, Chongqing, 400016 China; 3 The First People’s Hospital of Shuangliu District, Chengdu, Sichuan 610200 China

**Keywords:** Alzheimer’s disease, G protein-coupled receptor 43, Cyclophilin D, Neurons, Synapses, Mitochondria, Apoptosis

## Abstract

**Background:**

G protein-coupled receptor 43 (GPR43) is a critical signaling molecule involved in maintaining energy balance and immune homeostasis, making it a widely studied drug target. However, its role in Alzheimer’s disease (AD) remains unclear. This study aims to investigate the effects of GPR43 activation in an Aβ_1−42_-induced AD mouse model and to elucidate the underlying mechanisms.

**Methods:**

Experiments were performed using Aβ_1-42_-induced C57BL/6 mice (in vivo model) and the mouse hippocampal neuronal cell line HT22 (in vitro model). GPR43 gene expression and protein levels were analyzed in the brains of AD mice. Lentivirus-mediated GPR43 overexpression was employed to assess its effects on AD-like behaviors and pathological features. Cyclosporin A (CSA), a cyclophilin D (CypD) inhibitor, was used to investigate the pathological mechanisms of GPR43 in AD.

**Result:**

Compared to wild-type mice, GPR43 expression was downregulated in the cerebral cortex and hippocampus of Aβ_1-42_-induced AD mice and was primarily localized to neurons. GPR43 activation improved spatial learning and memory in AD mice. Furthermore, it upregulated the expression of brain-derived neurotrophic factor (BDNF), postsynaptic density protein 95 (PSD95), and synaptophysin (SYP), indicating enhanced neuronal and synaptic function. GPR43 upregulation also modulated the levels of mitochondrial damage-related enzymes, including superoxide dismutase (SOD), malondialdehyde (MDA), and lactate dehydrogenase (LDH) levels, and reduced mitochondrial swelling. Notably, GPR43 downregulated CypD protein levels, which are linked to mitochondrial permeability transition pore (mPTP) channels, thereby inhibiting apoptosis. Finally, in GPR43-knockdown cells, treatment with CSA significantly reduced the apoptosis rate, decreased BAX and Caspase-9 levels, and increased BCL-2 expression.

**Conclusion:**

GPR43 inhibits apoptosis in AD mice through the CypD signaling pathway, highlighting its potential as a novel target for drug development in AD treatment.

## Introduction

Alzheimer’s disease (AD) is a prevalent, chronic, and progressive neurodegenerative disorder that significantly contributes to dementia and ranks as the sixth leading cause of death among older adults (Apostolova 2016). The pathological hallmarks of AD include the accumulation of amyloid β (Aβ) protein, the formation of neurofibrillary tangles, and neuronal death (Selkoe 2011). Emerging evidence further implicates apoptosis and mitochondrial dysfunction in AD progression (Xu et al. 2022). Despite extensive research, the precise etiology of AD remains elusive, and no disease-modifying therapies are currently available. Although several drugs have been developed to reduce Aβ production or enhance its clearance in the brain, their efficacy in mitigating AD progression and cognitive decline remains inconclusive. Notably, contemporary drug discovery efforts are increasingly focused on novel therapeutic beyond the traditional Aβ and tau pathways (Cummings et al. 2023), offering renewed optimism for AD treatment through innovative strategies such as biologics and gene-editing technologies.

G protein-coupled receptors (GPRs), are seven-transmembrane domain receptors that specifically bind short-chain fatty acids (SCFAs) and are implicated in neurological diseases such as Parkinson’s disease (PD) and multiple sclerosis (MS) (Palczewski 2010). These receptors mediate cellular responses to hormones and neurotransmitters (Veenstra-VanderWeele et al. 2017; Shoichet and Kobilka 2012) and represent one of the most extensively studied classes of drug targets (Hauser et al. 2017). Among GPRs, GPR43 (also known as FFAR2) is uniquely activated by SCFAs and plays crucial roles in cellular homeostasis (Bindels et al. 2013), including regulation of energy metabolism, maintenance of calcium balance, modulation of oxidative stress, enhancement of insulin and hormone secretion, and support of adipocyte differentiation (Brown et al. 2003; Russo et al. 2021; Cox et al. 2009; Vinolo et al. 2011; Huang et al. 2020; Kimura et al. 2013; Miyamoto et al. 2019). Notably, GPR43 expression has been detected in the AD brain, with pronounced localization in the hippocampus (Wu et al. 2022). Razazan et al. demonstrated that GPR43 inhibition exacerbates Aβ-induced neurotoxicity, whereas its activation attenuates AD-associated pathological changes (Razazan et al. 2021). Despite these advances, the precise molecular mechanisms through which GPR43 influences pathogenesis of AD remain largely unexplored, necessitating further mechanistic interrogation.

Recent studies have identified cyclophilin D (CypD) as a critical regulator of the mitochondrial permeability transition pore (mPTP), whose pathological opening triggers mitochondrial swelling and subsequent release of pro-apoptotic factors such as cytochrome C. CypD is increasingly implicated in the pathogenesis of neurodegenerative disorders, including PD, MS and AD (Halestrap and Brenner 2003). The growing interest in CypD stems from its dual role as a peptidyl-prolyl isomerase within the mitochondrial matrix and a central modulator of mPTP dynamics, positioning it as a prime therapeutic target in mitochondrial medicine (Du et al. 2008). Mechanistically, CypD facilitates calcium overload-induced mPTP opening under pathological conditions, disrupting ATP synthesis and inducing mitochondrial dysfunction (Nakagawa et al. 2005). Notably, genetic ablation of CypD (CypD-KO models) confers neuroprotection by preserving mitochondrial integrity and bioenergetic capacity. Translational relevance is highlighted by recent clinical findings demonstrating that a novel CypD inhibitor rescues mitochondrial respiration and attenuates cognitive decline in patients with neurological diseases (Samanta et al. 2023). Collectively, these findings implicate CypD-mediated mPTP dysregulation as a pivotal contributor to AD progression through mechanisms related to mitochondrial dysfunction.

Since the role of GPR43 in AD remains unexplored-particularly its association with CypD-mediated apoptosis-this study aims to investigate the expression pattern of GPR43 in the brains of AD model mice. Specifically, we will examine its role in regulating learning and memory, mitochondrial and neuronal alterations, and synaptic plasticity. Furthermore, we seek to explore its potential relationship with cell apoptosis and the underlying molecular mechanisms.

## Methods and materials

### Animals

Eight-month-old male C57BL/6 mice (28–35 g, specific-pathogen-free [SPF]) were obtained from the Laboratory Animal Center of Chongqing Medical University, China. The mice were housed under controlled conditions (22 °C, 12-hour light/dark cycle) with ad libitum access to food and water. All experimental procedures were approved by the Animal Experimentation Ethics Committee of Chongqing Medical University (IACUC approval number: 2021–188) and conducted in accordance with international guidelines.

### AD model

#### Aβ_1-42_ preparation

Aβ_1−42_ (Sigma-Aldrich; A9810) powder was dissolved in 222 µl hexafluoroisopropanol (HFIP, Friendship gift) under a ventilated hood, followed by 15 min ultrasonication in a water bath. After solvent evaporation formed a transparent film, the peptide was resuspended in 22 µl dimethyl sulfoxide (DMSO) (Biosharp; BL165B) and 200 µl phosphate-buffered saline (PBS) (Biosharp; BL2214 A). The solution was then wrapped in aluminum foil and incubated at 4 °C for 7 days to allow fibril formation. Prior to use, the solution was centrifuged at 12,000×g for 10 min to remove pre-formed aggregates.

#### Modelling

Following intraperitoneal (i.p.) administration of pentobarbital sodium (50 mg/kg body weight), the depth of anesthesia was confirmed by the absence of pedal reflex and corneal response. The animals were then secured in a stereotaxic apparatus (RWD Life Science, Shenzhen, China), with body temperature maintained at 37 ± 0.5 °C using a feedback-controlled heating pad. Either Aβ_1−42_ (3 µg; 1 mg/ml) or an equivalent volume of saline (3 µl; Sham group) was slowly injected into the lateral ventricle. The injection needle was retained for 5 min before withdrawal to prevent backflow, and the surgical wound was sutured.

### Hippocampal lentivirus injection

Lentiviral vectors encoding GPR43-RNAi (LV-GPR43-RNAi) and control overexpression construct (LV-Con-OE) were designed and purchased from Tsingke Biological Technology. The anesthesia protocol matched that used during AD model induction. Using a 5-µl Hamilton syringe (Reno, NV, USA), 1.5 µL of LV-GPR43-RNAi, LV-Con-OE, or PBS (Sham group) was bilaterally injected into the dentate gyrus (DG) and Cornu Ammonis 1 (CA1) regions of the hippocampus at an infusion rate of 0.1 µl/min. Stereotaxic coordinates relative to bregma were: DG, (Anterior-Posterior [AP]:−2.0 mm; Medial-Lateral [ML]:−1.5 mm; Dorsal-Ventral [DV]:−2.0 mm) and CA1(AP: −2.0 mm; ML: −1.5 mm; DV: −1.5 mm) (Sada et al. 2015). The syringe was maintained in position for 10 min post-injection to minimize reflux. The same procedure was repeated on the contralateral hemisphere.

### Cell culture

The mouse hippocampal neuron cell line HT22 was maintained in Dulbecco’s modified Eagle medium (DMEM, 6124577; GIBCO, USA) supplemented with 10% fetal bovine serum (FBS, 086–150; wisent, Canada) and 1% penicillin-streptomycin (100 µg/ml) (Beyotime, C0222; China). Cells were cultured in an incubator (Thermo Fisher Scientific, MA, USA) at 37 °C under a humidified atmosphere with 5% CO_2_.

### Experimental design

The experiment included five in vivo groups: (i) 8-month-old wild-type mice (control); (ii) 8-month-old wild-type mice injected with PBS (Sham); (iii) 8-month-old AD mice induced by Aβ_1−42_ (AD); (iv) 8-month-old AD mice injected with control LV- GPR43-RNAi (Con-OE); and (v) 8-month-old AD mice injected with LV- GPR43-RNAi (GPR43-OE). In vitro, seven groups were established: (i) HT22 cells (control), HT22 cells with Si-CON and HT22 cells with Si-GPR43; (ii) Aβ_1−42_ induced HT22 cells (AD), Aβ_1−42_ induced HT22 cells with cyclosporin A (CSA), Aβ_1−42_ induced HT22 cells with Si-CON (AD/Si-CON), and Aβ_1−42_ induced HT22 cells with Si-GPR43 (AD/Si-GPR43). This study adhered to the principle of animal blinding, with researchers identifying mice solely by their ear tag numbers and remaining unaware of specific group assignments until statistical analysis.

### Western blotting

Cells and brain tissues (hippocampus and cortex) were harvested for western blot analysis (Qin et al. 2022). Protein extracts were resolved on 10% or 12.5% SDS–polyacrylamide gels and electrophoretically transferred onto 0.45-µm polyvinylidene difluoride (PVDF) membranes (Millipore, Billerica, MA, USA). The membrane was incubated at room temperature (RT) for 2 h in blocking buffer containing 5% (w/v) skim milk (Biofroxx, 1172GR100) prepared in Tris-buffered saline with 0.1% Tween-20 (Biofroxx,1247ML100) (TBST). Subsequently, membranes were incubated overnight at 4 °C with the following primary antibodies: rabbit anti-GPR43 (19952-1-AP; 1:1000), anti-glyceraldehyde-3-phosphate dehydrogenase (GAPDH) (60004-1-lg; 1:5000), anti-brain-derived neurotrophic factor (BDNF) (28205-1-AP;1:3000), anti-synaptophysin (SYP) (17785-1-AP; 1:50000), anti-cysteinyl aspartate specific proteinase 9 (Caspase 9) (10380-1-AP; 1:1000), anti-BCL2-Associated X (BAX) (50599-2-lg; 1:3000), anti-B-cell lymphoma/leukemia 2 (BCL-2) (26593-1-AP; 1:2000), and anti-CypD (12716-1-AP; 1:2000) from Proteintech in Wuhan, China, and anti- postsynaptic density protein 95 (PSD95) (AF300433; 1:1000) from AIFang Biological in Changsha, China. Membranes were washed three times with PBST (5 min per wash at 80 rpm) and incubated for 1 h with horseradish peroxidase (HRP)-conjugated goat anti-rabbit IgG secondary antibody (Biosharp; BL003 A; 1:10000) in TBST. After three additional PBST washes (20 min per wash at 120 rpm), protein bands were detected using an enhanced chemiluminescence (ECL) kit (Advansta, CA, USA) and imaged with a ChemiDoc Touch Imaging System (Bio-Rad, CA, USA). Protein expression levels were quantified using Image Lab software (version 6.1; Bio-Rad, CA, USA) with GAPDH serving as the loading control.

### Quantitative reverse-transcription polymerase chain reaction (qRT-PCR)

Total RNA was isolated from HT22 cells and brain tissues reagent according to the manufacturer’s protocol (Mei5 Biotech, Beijing, China). qRT-PCR was performed on an Eco Real-Time PCR system (Illumina, CA, USA) with 5x M5 HiPer SYBR Premix EsTaq (with Tli RNase H) in 10 µl reactions volumes containing 20 ng of RNA template. The primer sequences used were: GAPDH: forward: 5′-ACC ACA GTC CAT GCC ATC AC-3′) and reverse (5′-TCC ACC CTG TTG CTG TA-3′); GPR43: forward (5′-AGA AGG AAT GGC TAC CCT-3′) and reverse (5′- TTA GAG CTT TCC CGG CAT TC-3′); CypD: forward (5- GCC CTA TGC ACT GGT GAG AA-3′) and reverse (5′-CTC CTG TGC CAT TGT GGT TG-3′). Relative mRNA expression levels of GPR43 and CypD were calculated using the 2 − ΔΔCt method with GAPDH as the endogenous control. Data were normalized to the control group values (set as 1) within each experiment.

### Behavioral testing

Morris Water Maze (MWM) (Gehring et al. 2015): The apparatus consisted of a circular pool (170 cm diameter × 50 cm height) filled with opaque water (23 ± 1 °C; 30 cm depth) divided into four virtual quadrants (east, west, south, and north). A transparent acrylic escape platform (14 cm diameter × 29 cm height) was submerged 1 cm below the water surface in the target quadrant and remained stationary throughout the 5-day acquisition phase. The protocol included two phases:

1. Training Phase (Days 1–5): Mice underwent four trails daily (inter-trail interval: 30 min), staring randomly from different quadrants with 60 s maximum trail duration. Animals failing to find the platform within 60 s were gently guided to it and allowed 15 s of spatial orientation.
2. Test Phase (Day 6): The platform was removed, and mice were released from the quadrant opposite to the original target location. Behavior was recorded for 60 s using an automated tracking system (SMART v3.0 software).

Video recordings were scored by two blinded experimenters, measuring: Latency Target, Latency 1 st Entrance to Target, Target Crossings and Time in zoo.

Y-Maze Spatial Memory Test (Rao et al. 2021; Chesworth et al. 2012): This study evaluated rodents’ spatial working memory and exploratory behavior using a standardized Y-maze protocol. The apparatus consisted of three identical arms (40 × 10 × 15 cm; 120° inter-arm angles) constructed from gray polyvinyl chloride. Behavioral testing was performed under controlled conditions (50 lx illumination, 60 dB white noise background). Experiment Protocol: (1) Habituation Phase: one arm (designated as the novel arm) was blocked, permitting 10 min exploration of the remaining two arms. (2) Test Phase: Following a 2-h inter-trail interval in home cages, all arms became accessible for 10 min free exploration.

Video recordings were scored by two blinded experimenters, measuring: Time in novel arm, Spontaneous Alternation ration (%), and Total arm entries.

### Immunofluorescence staining (IF)

The procedure was performed according to established methods in our laboratory (Xiong et al. 2016). After thawing the frozen sections for 1 h at RT, the samples were washed three times with 0.01 M PBS (5 min per wash). For non-GPR43 protein targets, tissue permeabilization was performed by incubating sections with 0.4% Triton X-100 (Beyotime, P0096) in PBS for 60 min at RT, followed by three additional PBS washes (5 min each). Antigen retrieval was performed using sodium citrate buffer (pH 6.0) with microwave heating for 3 min. Following cooling to room temperature, the sections were washed three times with PBS (5 min per wash). Non-specific binding sites were blocked by incubating the sections with 10% (v/v) normal goat serum (Boster, AR0009) for 2 h at RT. The sections were then incubated overnight at 4 °C with a mixture of primary antibodies, followed by PBS washes to remove unbound antibodies. Subsequently, sections were incubated with secondary fluorophore-conjugated antibodies (1 h at RT, light-protected). The primary antibodies used included rabbit anti-GPR43 (Proteintech;19952-1-AP; 1:200), rabbit anti-SYP (Proteintech;17785-1-AP; 1:200), mouse anti-microtubule-associated protein 2 (MAP2) (Proteintech;67015-1-Ig; 1:200), mouse anti-glial fibrillary acidic protein (GFAP) (Proteintech;66827-1-lg; 1:200), and mouse anti-ionized calcium-binding adapter molecule 1(Iba1) (Proteintech;60190-1-lg; 1:200). The secondary antibodies were Dylight 549 conjugated Goat Anti-Rabbit IgG (H + L) (Abbkine; A23320; 1:100) and donkey anti-rabbit Alexa Fluor 488 (Boster; BA1145; 1:100). Images were captured using a fluorescence microscope (Nikon Eclipse C1, Tokyo, Japan). Fluorescence colocalization analyses were conducted with Image J.

### Immunohistochemistry staining (IHC)

We analyzed coronal sections of the prefrontal cortex (PFC) (AP: +2.70 to + 1.98 mm relative to Bregma, ML: ± 0.35, DV: −1.85 mm; 40 μm-thick) due to its critical roles in higher cognition (e.g., decision-making, emotional regulation) and vulnerability in neurodegenerative diseases (Tang et al. 2022; Cavanagh et al. 2018; Huda et al. 2020; Butt and Lak 2020; Stokes 2015). Coordinates followed Paxinos and Franklin (4 th ed.) (Chon et al. 2019).The IHC protocol comprised these sequential steps: After dewaxing, hydration, the tissue Sect. (5 μm thick) underwent antigen retrieval in citrate buffer (pH 6.0) using microwave heating (8 min boiling, 8 min resting, 7 min low-medium power). After cooling to room temperature (RT), sections were washed in PBS (3 × 5 min). Endogenous peroxidase activity was blocked with 3% hydrogen peroxide (25 min, RT, dark), followed by PBS washes (3 × 5 min). Non-specific binding was blocked with 3% BSA (Servicebio; G5001) (30 min, RT). Primary antibody incubation was performed overnight at 4 °C. The next day, sections were washed and incubated with HRP-conjugated secondary antibody (60 min, RT). DAB (Servicebio; G1211) chromogenic development was monitored microscopically. Sections were counterstained with Hematoxylin (Servicebio; G1004) for 3 min, followed by differentiation (Servicebio;1309) and bluing (Servicebio;1340). Sections were dehydrated through graded alcohols, cleared in xylene, and cover-slipped with neutral balsam. Images were captured with an optical light microscope (Nikon Eclipse E100, Tokyo, Japan) at 400× magnification. Positive immunoreactivity was identified by brownish-yellow DAB precipitation, while nuclei appeared blue with hematoxylin counterstain.

### Hematoxylin-eosin staining (H&E)

Serial paraffin sections (5 μm thick) of each sample were cut with a microtome. Following dewaxing, hydration, and staining with hematoxylin and eosin (Beyotime Biotechnology, Shanghai, China), images were captured with an optical light microscope (Nikon Eclipse E100, Tokyo, Japan) at×400 magnification. Neurons were counted if they exhibited: (i) Distinct nucleoli, and (ii) Intact cellular morphology. Two blinded researchers independently counted three non-overlapping regions of the PFC and hippocampus on the same slice using ImageJ software (400× magnification, 0.2 mm²/field).

### Nissl staining

Paraffin-embedded tissues were sectioned at 5 μm thickness using a microtome. Following dewaxing and hydration, sections were stained with toluidine blue (Servicebio; G1032) (pH 4.5) for 5 min at RT, followed by differentiated in 0.1% acetic acid (Servicebio;10004160). After rinsed, sections were dehydrated through graded ethanol, cleared in xylene, and mounted with neutral balsam. Stained sections were imaged under a light microscope (Nikon Eclipse E100; 400× magnification). Image selection criteria required: (i) well-defined Nissl bodies (purple cytoplasmic granules), and (ii) precipitation-free background staining. Nissl + Cell Counting: Positivity threshold: ≥3 distinct cresyl violet-stained Nissl bodies per cell. Two blinded researchers independently counted three non-overlapping regions of the PFC and hippocampus on the same slice using ImageJ software (400× magnification, 0.2 mm²/field).

### Golgi staining

To investigate the effect of GPR43 on dendritic formation in vivo, we performed Golgi staining using a GolgiStain Kit (Servicebio; G1069). Briefly, brain tissues were immersed in Golgi staining solution for 14 days and stored in a cool, ventilated environment. The tissues were then transferred to 80% glacial acetic acid (Sinopharm Chemical Reagent, 10000218) overnight until softened, followed by immersion in 30% sucrose solution (Sinopharm Chemical Reagent, 57-50-1). Using a cryostat microtome (CRYOSTAR NX50, Thermo, MA, USA), 100 μm sections were cut and mounted on gelatin-coated slides, which were kept in darkness overnight. After drying, sections were sequentially treated with: (Apostolova 2016) concentrated ammonia solution (Sinopharm Chemical Reagent, 10002118) for 15 min (Selkoe 2011), acidic film fixing solution for 15 min, and (Xu et al. 2022) distilled water for 3 min before air-drying and cover slipping. Dendritic morphology was visualized under bright-field illumination using an upright microscope (ECLIPSE E100, Nikon, Tokyo, Japan).

### Mitochondrial function experiment

#### Transmission electron microscopy (TEM)

Animals were perfused using the standard perfusion method (Awasthi et al. 2021). After removing the brain, hippocampal tissue was quickly cut into 1 mm^3^ pieces with a sharp blade and fixed in 4% paraformaldehyde for 2–3 h. The tissue was then sliced into 1 × 1 mm pieces, stained with lead citrate for 5 min and uranyl acetate for 30 min, followed by thorough rinsing and drying. Electron microscopy was performed at 60 kV using a Philips Morgagni TEM equipped with a CCD, capturing images at magnifications above 20,000 magnifications.

#### Superoxide dismutase (SOD), malondialdehyde (MDA), and lactate dehydrogenase (LDH) assays

The mouse brain tissues was rinsed with PBS and gently dried using filter paper. The appropriate brain tissue was weighed and homogenized in an ice bath at a mass-to-extraction solution ratio of 1:10. The supernatant was collected after centrifugation at 8,000 g for 10 min at 4 °C. Levels of SOD, MDA, and LDH were determined using respective kits: SOD (Solarbio; BC0175), MDA (Solarbio; BC0020) and LDH (Solarbio, BC0685).

### Cell counting kit-8 (CCK-8)

To elucidate GPR43-mediated AD pathogenesis via the CypD pathway, we employed cyclosporin A (Sigma-Aldrich; SML1018-1ML), a specific CypD inhibitor. Cell seeding: HT22 cells were plated at 5 × 10^3^ cells/well in 96-well plates. Following the manufacturer’s instructions, cells was treated with CCK-8(Biosharp; BS350B) reagent and incubated for 2 h at 37 °C in 5% CO₂. Absorbance was measured at 450 nm using enzyme-linked immunosorbent assay (ELISA) instrument. The calculated values served as the final experimental, which were saved for statistical analysis.

### Statistical analysis

Data analysis was performed using GraphPad Prism 8 software (GraphPad Software Inc., La Jolla, CA, USA) or SPSS 20.0. Data was presented as mean ± SEM. The normality of continuous variables was assessed using the Shapiro-Wilk test. For normality distribution data, a one-sample *t*-test was employed to compare two groups, both Bartlett’s test (for homogeneity of variance) and one-way or two-way ANOVA were used for multi-group comparisons, followed by Dunnett’s T3 post hoc test. If normality was not achieved, the Wilcoxon Signed Rank Test was utilized for within-group comparisons and the Kruskal-Wallis test for multi-group comparisons. Statistical significance was set at * *p* < 0.05, ** *p* < 0.01, *** *p* < 0.0001.

## Results

### GPR43 expression and distribution in AD mouse brains

**Fig. 1:**
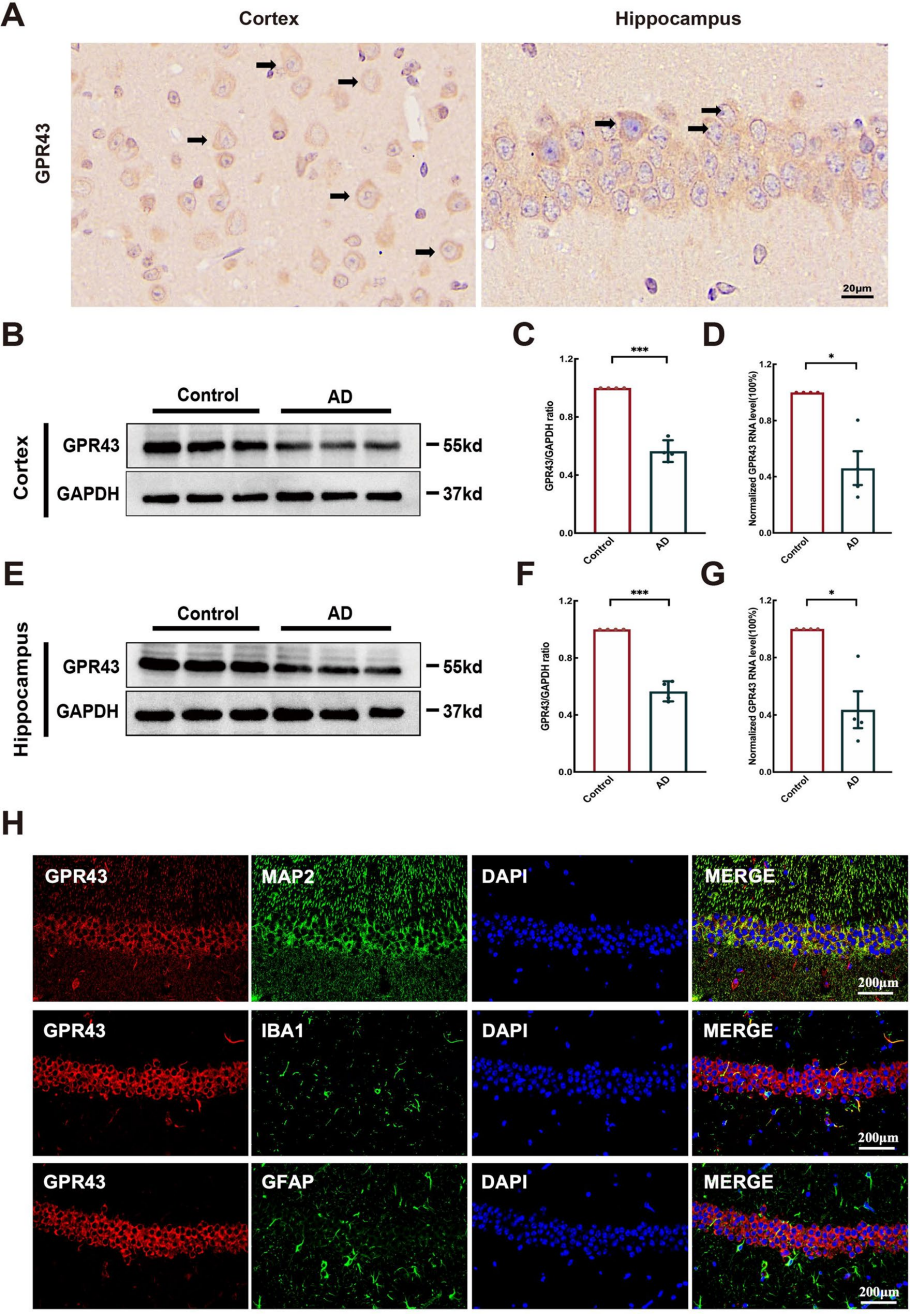
GPR43 expression was significantly reduced in the brains of Aβ_1−42_ model mice and was predominantly localized to neurons. **A** Immunohistochemical staining of GPR43 in the cortex and hippocampus (Scale bar: 20 μm). **B** Western blot analysis of GPR43 in the cerebral cortex. **C** Quantification of GPR43 protein expression in the cortex. **D** Quantitative reverse-transcription polymerase chain reaction (qRT‒PCR) analysis of GPR43 mRNA levels in cortical tissues. **E** Western blot analysis of GPR43 in hippocampal tissues. **F** Quantitative analysis of GPR43 mRNA levels in the hippocampus. **G** qRT‒PCR analysis of GPR43 mRNA levels in hippocampal tissues. **H** Immunofluorescence showing GPR43 (red) colocalization with neuronal marker microtubule-associated protein 2 (MAP2) (green) but not with microglial marker ionized calcium-binding adapter molecule 1(IBA1) or astrocytic marker glial fibrillary acidic protein (GFAP) (green) in hippocampal tissues. Nuclei (blue) were counterstained with DAPI (blue) (Scale bar: 200 μm). Data were presented as mean ± SEM (*n* = 4). *p < 0.05, **p < 0.01, and ***p < 0.001

The immunohistochemical staining demonstrated GPR43 expression in both cortical and hippocampal tissues of AD mouse brains (Fig. 1A). Western blotting and qRT-PCR analyses revealed statistically significant decrease in GPR43 expression in the AD group compared to controls, in both cortex (Western blot: 0.57 ± 0.04; qRT-PCR: 0.46 ± 0.12) and hippocampus (Western blot: 0.58 ± 0.04; qRT-PCR: 0.44 ± 0.13) (Fig. 1B-G). Immunofluorescence analysis showed that GPR43 colocalized with neuronal markers (MAP2) but not with astrocytic (GFAP)or microglial (IBA1) in both hippocampal CA1 and cortical regions (Fig. 1H). These findings suggest that GPR43 may play a crucial role in AD pathogenesis.

### Effect of GPR43 overexpression on behavioral phenotypes in AD mice

**Fig. 2:**
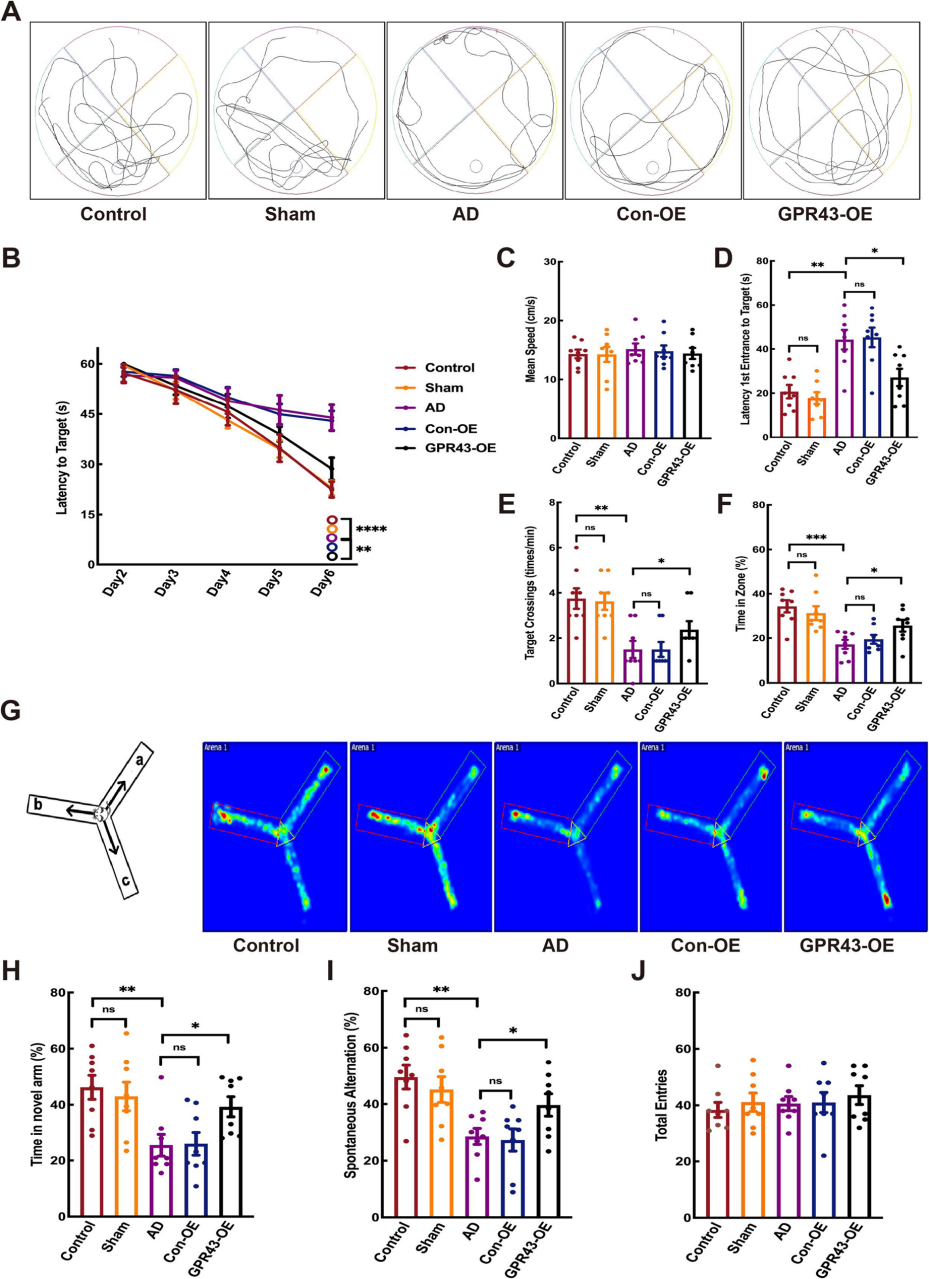
GPR43 overexpression improved cognitive function in the Alzheimer’s disease (AD) model mice.  Morris Water Maze (MWM) test: **A** Representative swimming trajectories (test phase). **B** Escape latency during training trails. **C** Average swimming speed during training. **D** First latency to enter the target quadrant (test phase). **E** Number of target quadrant crossings (test phase). **F** Time spent in the target quadrant (test phase). Y-Maze test: **G** Heatmap of movement trajectories. **H** Total arm entries. **I** Spontaneous alternation rate (%). **J** Percentage time spent in the novel. Data were presented as the mean ± SEM (*n* = 8). *p < 0.05, **p < 0.01, and ***p < 0.001

The MWM trajectory maps effectively illustrated swimming paths during testing (Fig. 2A). Escape latency decreased across all groups during training. On day 6, the GPR43-OE group showed significantly shorter latency to target time than the AD group (50.3 ± 2.550 s vs. 45.69 ± 5.493 s; *p* < 0.05; Fig. 2B). No significant differences in swimming speed were observed among groups (control: 14.25 ± 0.86 cm/s; Sham group:14.12 ± 1.45 cm/s; AD: 15.31 ± 1.07 cm/s; Con-OE group: 14.93 ± 1.11 cm/s; GPR43-OE group: 14.70 ± 1.02 cm/s; *p* > 0.05; Fig. 2C). During testing phase, the GPR43-OE group demonstrated a shorter time of latency 1 st entrance to target (27.14 ± 3.92 s vs. AD: 44.23 ± 4.41 s; *p* < 0.05; Fig. 2D), more target quadrant crossings (2.38 ± 0.38 vs. AD:1.50 ± 0.33; *p* < 0.05; Fig. 2E), and longer percentage target area time (25.63 ± 2.70% vs. AD:17.20 ± 2.04%; *p* < 0.05; Fig. 2F). No significant difference was observed between control/sham groups or AD/Con-OE groups (*p* > 0.05, Fig. 2B-F).

Y-maze heat maps accurately depicted the exploration trajectories (Fig. 2G). The GPR43-OE group showed: longer novel arm exploration time (39.29 ± 3.58 s vs. AD: 25.52 ± 3.93 s; *p* < 0.05; Fig. 2H) and higher spontaneous alternation rate (39.68 ± 3.96% vs. AD:28.58 ± 2.81%; *p* < 0.05; Fig. 2I). No significant differences were observed in: spontaneous alternation (control: 49.55 ± 4.23% vs. Sham:45.13 ± 4.55%; AD:28.58 ± 2.81% vs. Con-OE: 27.31 ± 3.93%) and novel arm preference (control:46.23 ± 4.31% vs. Sham: 42.99 ± 5.08 s, and AD: 25.52 ± 3.93% vs. Con-OE: 26.00 ± 4.10%) (all *p* < 0.05; Fig. 2H, I). The total entries showed no group difference (control: 38.38 ± 2.68; Sham:41.13 ± 3.27; AD:40.63 ± 2.50; Con-OE: 41.00 ± 3.57; GPR43-OE: 43.63 ± 3.33; *p* > 0.05; Fig. 2J).

### GPR43 alleviated neuronal damage in AD model mice

**Fig. 3:**
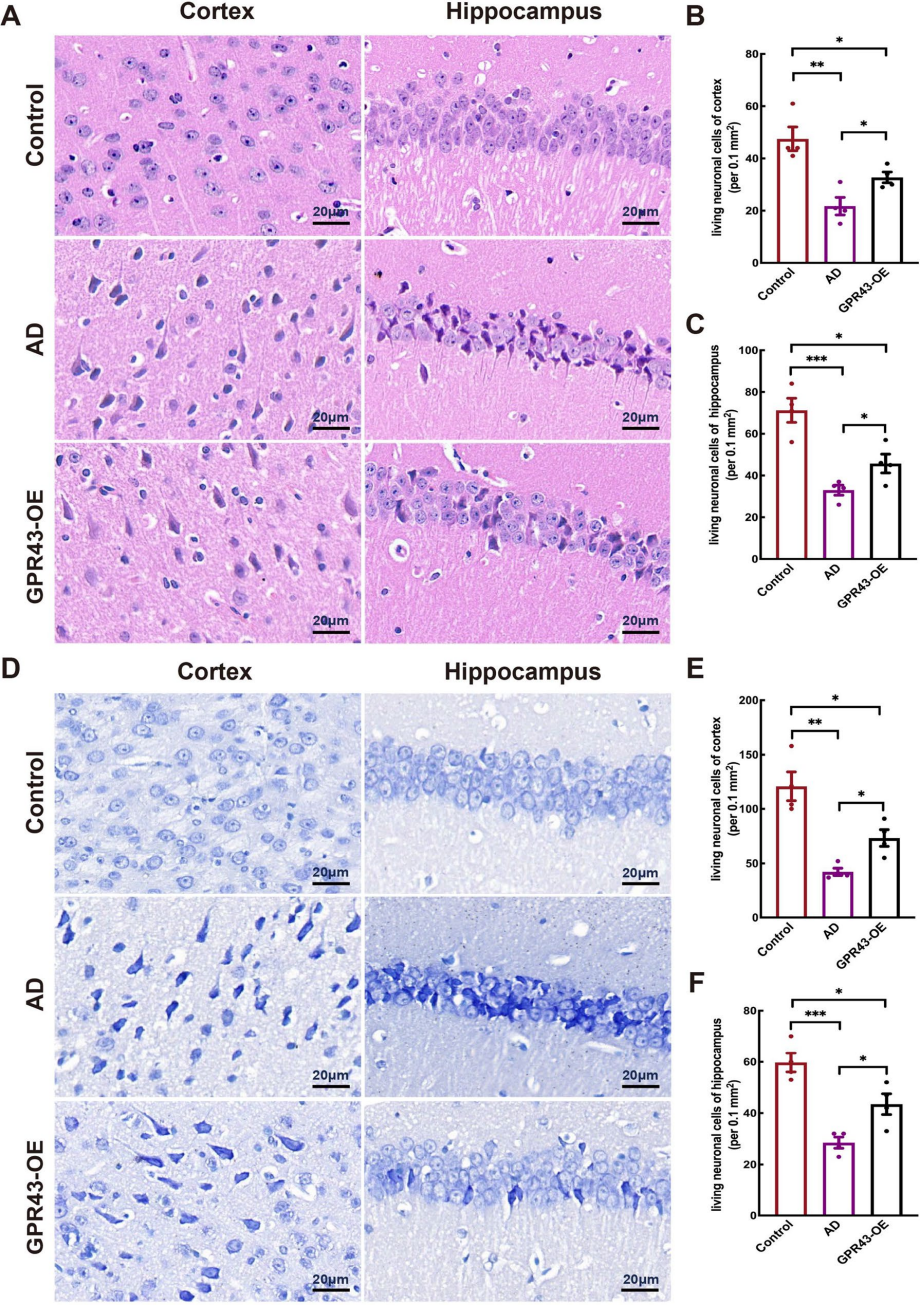
GPR43 overexpression attenuated neuronal damage in Alzheimer’s disease (AD) model mice. **A** Hematoxylin-eosin (H&E) staining in the cortex and hippocampus. Quantitative analysis of viable cells in the (**B**) cortex and (**C**) hippocampus. **D** Nissl staining of cortical and hippocampal neurons. Quantification of surviving neuron in the (**E**) cortex and (**F**) hippocampus. Scale bar: 20 μm. Data were presented as the mean ± SEM (*n* = 4). *p < 0.05, **p < 0.01, and ***p < 0.001

**Fig. 4:**
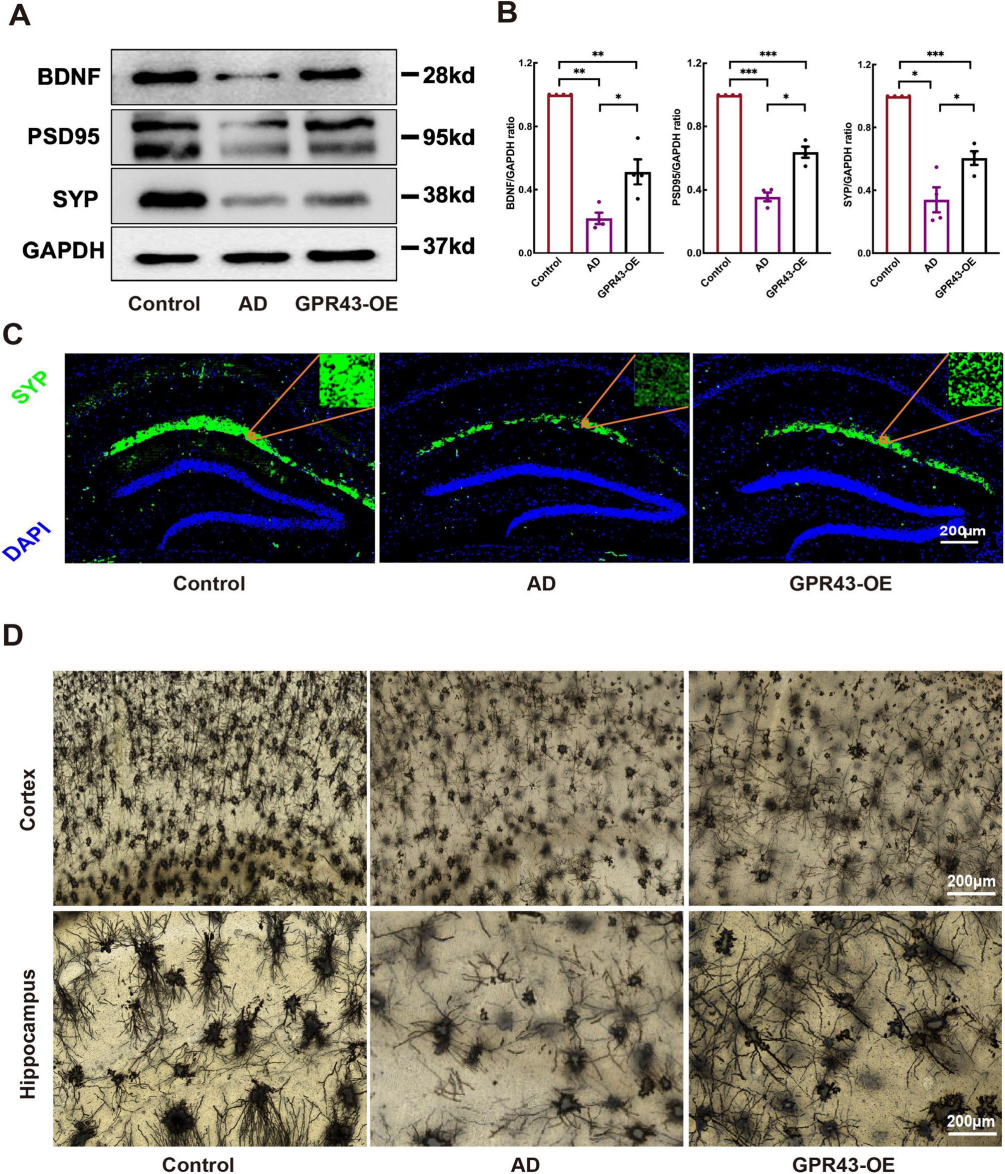
GPR43 overexpression promoted structural plasticity of dendritic spines in cortical neurons. **A** Western blot analysis of neurotrophic factor (brain-derived neurotrophic factor [BDNF]) and synaptic proteins (postsynaptic density protein 95[PSD95]) and synaptophysin [SYP]) in the hippocampal tissues. **B** Quantitative analysis of BDNF, PSD95, and SYP protein levels. **C** Immunofluorescence staining of SYP (green) in the hippocampus; nuclei were counterstained with DAPI (blue) (Scale bar: 200 μm). **D** Representative images of Golgi-Cox staining in cortical and hippocampal tissues (Scale bar:200 μm). Data were presented as the mean ± SEM (*n* = 4). *p < 0.05, **p < 0.01, and ***p < 0.001

H&E and Nissl staining assessed neuronal integrity across groups (Fig. 3A and D). Quantitative analysis showed: GPR43-OE group displayed intermediate living number cells in both cortex (control: 47.50 ± 4.56; AD:21.75 ± 3.35; GPR43-OE: 32.75 ± 2.06; *p* < 0.05; Fig. 3B) and hippocampus (control: 71.25 ± 5.79; AD:33.00 ± 2.42; GPR43-OE:45.75 ± 4.50; *p* < 0.05; Fig. 3C) by H&E staining. In contrast, GPR43-OE revealed a higher count of living neurons vs.AD (control:120.80 ± 13.22; AD:42.25 ± 3.35; GPR43-OE:73.25 ± 15.37; *p* < 0.05; Fig. 3E) in cortex and vs.AD (control:59.75 ± 3.71; AD:28.50 ± 2.18; GPR43-OE:43.50 ± 4.05; *p* < 0.05; Fig. 3F) in hippocampus by Nissl staining. Additionally, BDNF protein levels, reflective of neuronal nutritional status, were significantly elevated in the GPR43-OE group vs.AD (0.51 ± 0.08 vs. AD:0.22 ± 0.04; *p* < 0.05; Fig. 4A and B). Collectively, these results demonstrated GPR43 overexpression attenuates neuronal degeneration.

### GPR43 promoted dendritic spine remodeling in AD model mice

The synaptic proteins PSD-95 and SYP were quantified as functional readouts of synaptic integrity. Western blot analyses revealed significant upregulation of both PSD-95 (0.49 ± 0.10 vs. 0.22 ± 0.04 in AD, *p* < 0.05) and SYP (0.61 ± 0.04 vs. 0.34 ± 0.08 in AD; *p* < 0.05) in the whole brain lysates from GPR43-overexpressing (GPR43-OE) mice compared to AD littermates (Fig. 4A and B). Immunofluorescence intensity analysis showing increasing brightness of SYP in the GPR43-OE group relative to the AD group (Fig. 4C). Moreover, Golgi-Cox staining demonstrating increased spine density in cortical and hippocampal neurons of GPR43-OE mice versus AD and controls (Fig. 4D).

### GPR43 overexpression improved mitochondrial function in AD mice

**Fig. 5:**
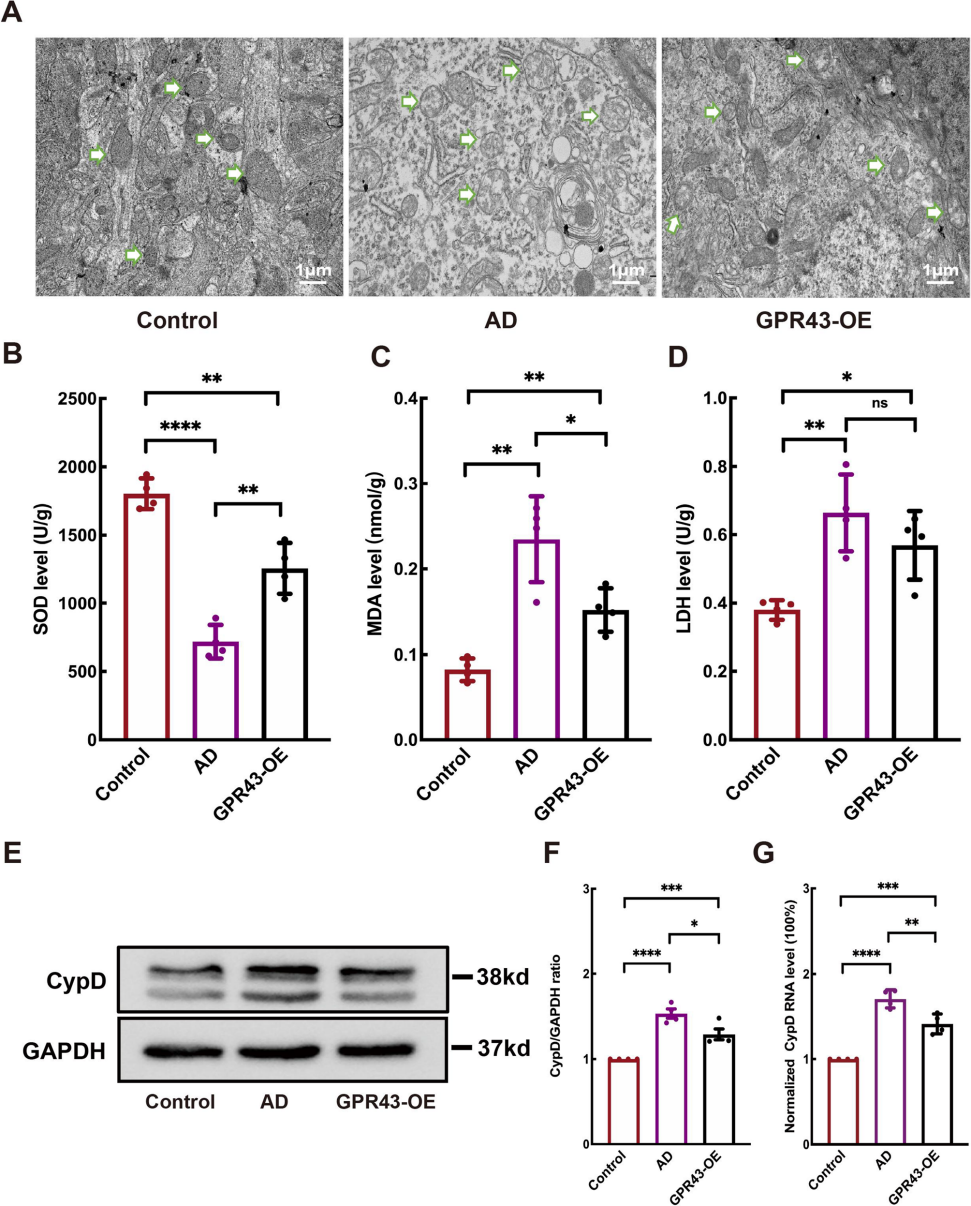
GPR43 overexpression attenuated mitochondrial dysfunction in Alzheimer’s disease (AD) model mice. **A** Representative electron micrographs showing mitochondrial swelling in hippocampal neurons. Quantitative analysis of (**B**) superoxide dismutase (SOD), (**C**) malondialdehyde (MDA), and (**D**) lactate dehydrogenase (LDH) levels. **E** Western blot analysis of cyclophilin D (CypD) expression in hippocampal tissues. **F** Quantification of CypD protein levels. **G** qRT-PCR analysis of CypD mRNA expression. Scale bar:1 μm. Data were presented as the mean ± SEM (*n* = 4). *p < 0.05, **p < 0.01, ***p < 0.001, and ****p < 0.0001

Ultrastructural analysis via electron microscopy demonstrated that mitochondria in the GPR43-OE group exhibited significantly attenuated swelling compared to those in the AD group (Fig. 5A). Biochemical analyses demonstrated that SOD activity was markedly elevated in the GPR43-OE group (1258.00 ± 92.92 U/g) relative to AD group (718.10 ± 61.34 U/g; *p* < 0.05; Fig. 5B). Conversely, the GPR43-OE group exhibited improved redox homeostasis, as evidenced by decreased MDA levels (0.15 ± 0.01 nmol/g vs. AD:0.23 ± 0.03 nmol/g; *p* < 0.05; Fig. 5C) and reduced lactate LDH release (0.57 ± 0.05 U/g vs. AD:0.66 ± 0.06 U/g; *p* < 0.05; Fig. 5D). At the molecular level, both Western blotting and qRT-PCR analyses confirmed downregulation of CypD expression in GPR43-OE group, with protein levels decreasing from 1.53 ± 0.05 to 1.29 ± 0.06; *p* < 0.05; Fig. 5E), and mRNA expression declining from 1.71 ± 0.05 to 1.41 ± 0.06; *p* < 0.05; Fig. 5G) compared to AD group, collectively indicating enhanced mitochondrial integrity through GPR43 overexpression.

### GPR43 overexpression reduced apoptotic bodies in AD mice

**Fig. 6:**
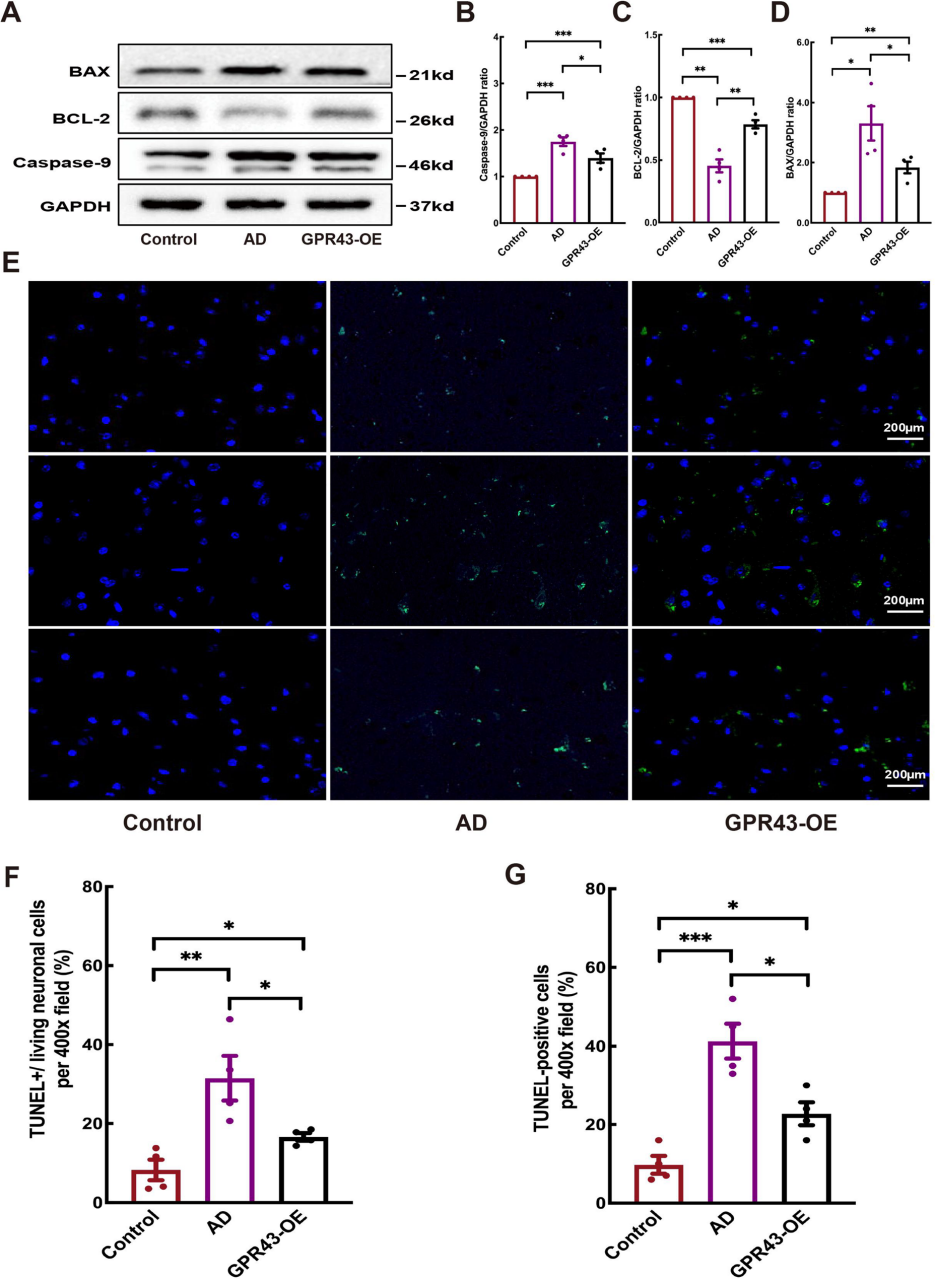
GPR43 overexpression suppressed neuronal apoptosis in Alzheimer’s disease (AD) model mice. **A** Western blot analysis of apoptosis-related proteins in brain tissues. Quantification of (**B**) BCL2-Associated X (BAX), (**C**) B-cell lymphoma/leukemia 2 (BCL-2), and (**D**) cleaved Caspase-9 protein levels (normalized to GAPDH). **E** Representative TUNEL (TRITC, green) staining in brain sections (nuclei counterstained with DAPI, blue). Scale bar: 200 μm. Quantitative analysis of (**F**) TUNEL^**+**^**/**-living neuronal cells and (**G**) TUNEL-positive neurons. Data were presented as the mean ± SEM (*n* = 4). *p < 0.05, **p < 0.01, and ***p < 0.001

Figure 6A displayed the expression patterns of key apoptosis-related proteins in different experimental groups. Western blot analysis demonstrate that Caspase-9 levels were significantly elevated in the GPR43-OE group (1.40 ± 0.10) compared to the AD model group (1.75 ± 0.09; *p* < 0.05; Fig. 6B). Notably, we observed a marked increase in the anti-apoptotic protein BCL-2 in the GPR43-OE group (0.79 ± 0.03) relative to AD controls (0.45 ± 0.05; *p* < 0.05; Fig. 6C), accompanied by a concurrent decrease in the pro-apoptotic protein BAX (GPR43-OE:1.84 ± 0.19 vs. AD:3.31 ± 0.57; *p* < 0.05; Fig. 6D). Complementary TUNEL assays revealed substantial reductions in neuronal apoptosis, with the GPR43-OE group showing fewer TUNEL+/living neurons cells (16.62 ± 0.95 vs. AD: 31.45 ± 5.67; *p* < 0.05; Fig. 6F) and lower percentages of TUNEL-positive cells (22.75 ± 2.926 vs. AD:41.25 ± 4.44; *p* < 0.05; Fig. 6G) per 100x microscopic field, collectively indicating the anti-apoptotic effects of GPR43 overexpression in AD models.

### GPR43 silencing elevated apoptotic rate in Aβ1–42-treated HT22 cells

**Fig. 7:**
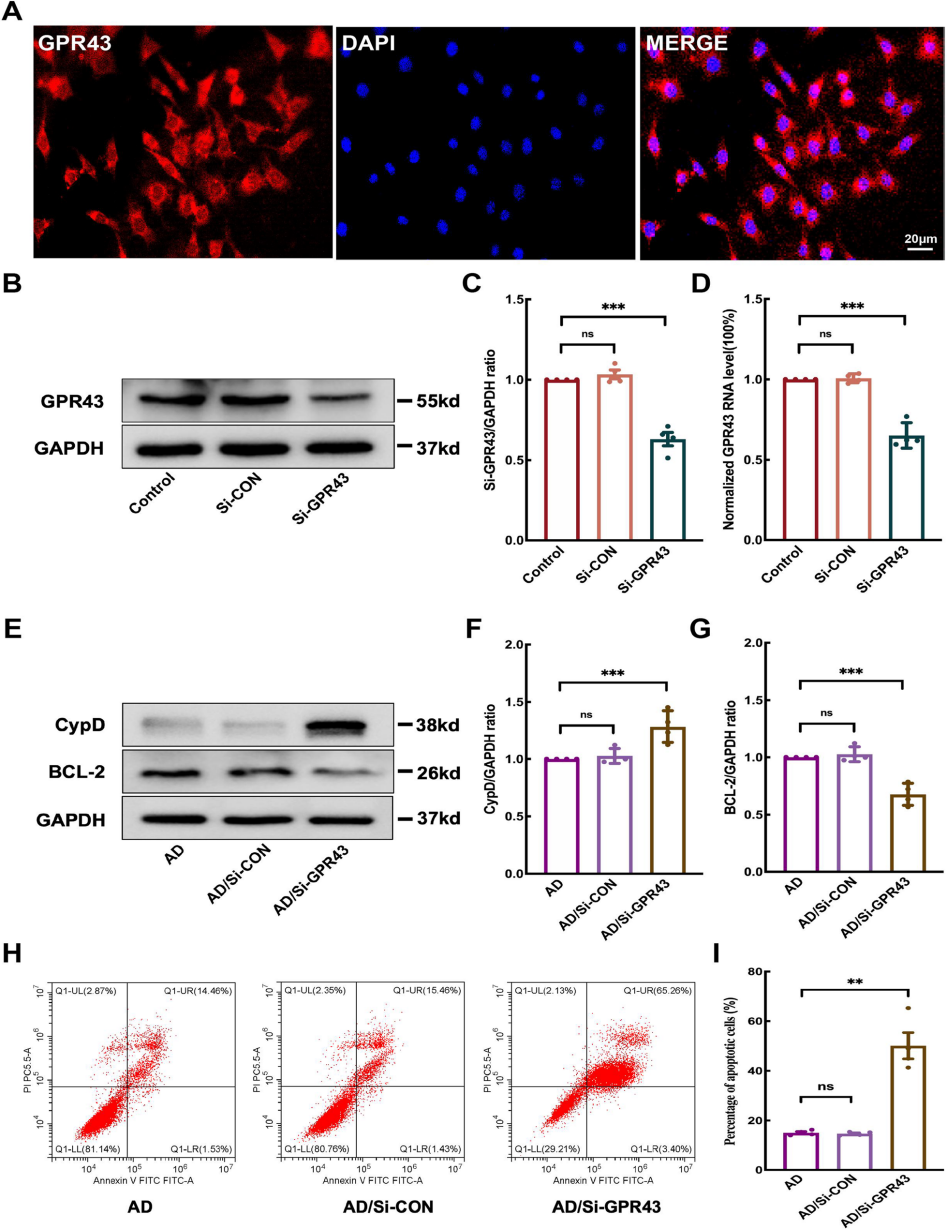
Si-GPR43 exacerbated Aβ_1–42_-induced apoptosis in HT22 cells. **A** Immunofluorescence showing GPR43 (red) localization on HT22 cell membranes (nuclei counterstained with DAPI, blue). Scale bar: 20 μm. **B** Western blot confirmation of successful GPR43 knockdown. **C** Quantification of GPR43 protein expression levels. **D** qRT-PCR analysis of GPR43 mRNA levels in knockdown cells. **E** Western blot analysis of cyclophilin D (CypD) and B-cell lymphoma/leukemia 2 (BCL-2) expression. Quantitative analysis of (**F**) CypD and (**G**) BCL-2 protein levels. **H** Representative flow cytometry plots of apoptotic cells. **I** Quantification of apoptosis rates. Data were presented as the mean ± SEM (*n* = 4). **p < 0.01, ***p < 0.001, and ****p < 0.0001

To elucidate the functional role of GPR43 in AD pathogenesis, we performed recovery experiments using the HT22 murine hippocampal neuronal cell line. Immunofluorescence analysis confirmed membrane localization of GPR43 in these cells (Fig. 7A). Successful establishment of a GPR43 knockdown model was verified through siRNA-mediated silencing, with Western blot analysis showing significant reduction in GPR43 protein expression (0.63 ± 0.04 in Si-GPR43 vs. 1.03 ± 0.03 in Si-CON and 1.0 ± 0 in control; *p* < 0.05; Fig. 7C) and qRT-PCR demonstrating corresponding mRNA downregulation (0.65 ± 0.04 in Si-GPR43 vs.1.01 ± 0.01 in Si-CON and 1.0 ± 0 in control; *p* < 0.05; Fig. 7D). Notably, GPR43 knockdown in Aβ1–42-treated cells resulted in dysregulation of apoptotic mediators, showing elevated CypD expression (1.29 ± 0.07 in AD/Si-GPR43 vs. 1.03 ± 0.03 in AD/Si-CON and 1.0 ± 0 in AD; *p* < 0.05; Fig. 7F) and increased BCL-2 levels (0.68 ± 0.05 in AD/Si-GPR43 vs. 1.01 ± 0.02 in Si-CON and 1.0 ± 0 in AD; *p* < 0.05; Fig. 7G). Flow cytometry analysis revealed a 3.0-fold increase in apoptotic cells in GPR43-depleted AD models (50.13 ± 5.29% in AD/Si-GPR43 vs. 15.07 ± 0.51% in AD; *p* < 0.05; Fig. 7I), collectively demonstrating that GPR43 suppression exacerbates apoptotic vulnerability in Aβ_1–42_-challenged hippocampal neurons.

### GPR43 regulated the pathogenesis of AD through the CypD pathway

**Fig. 8:**
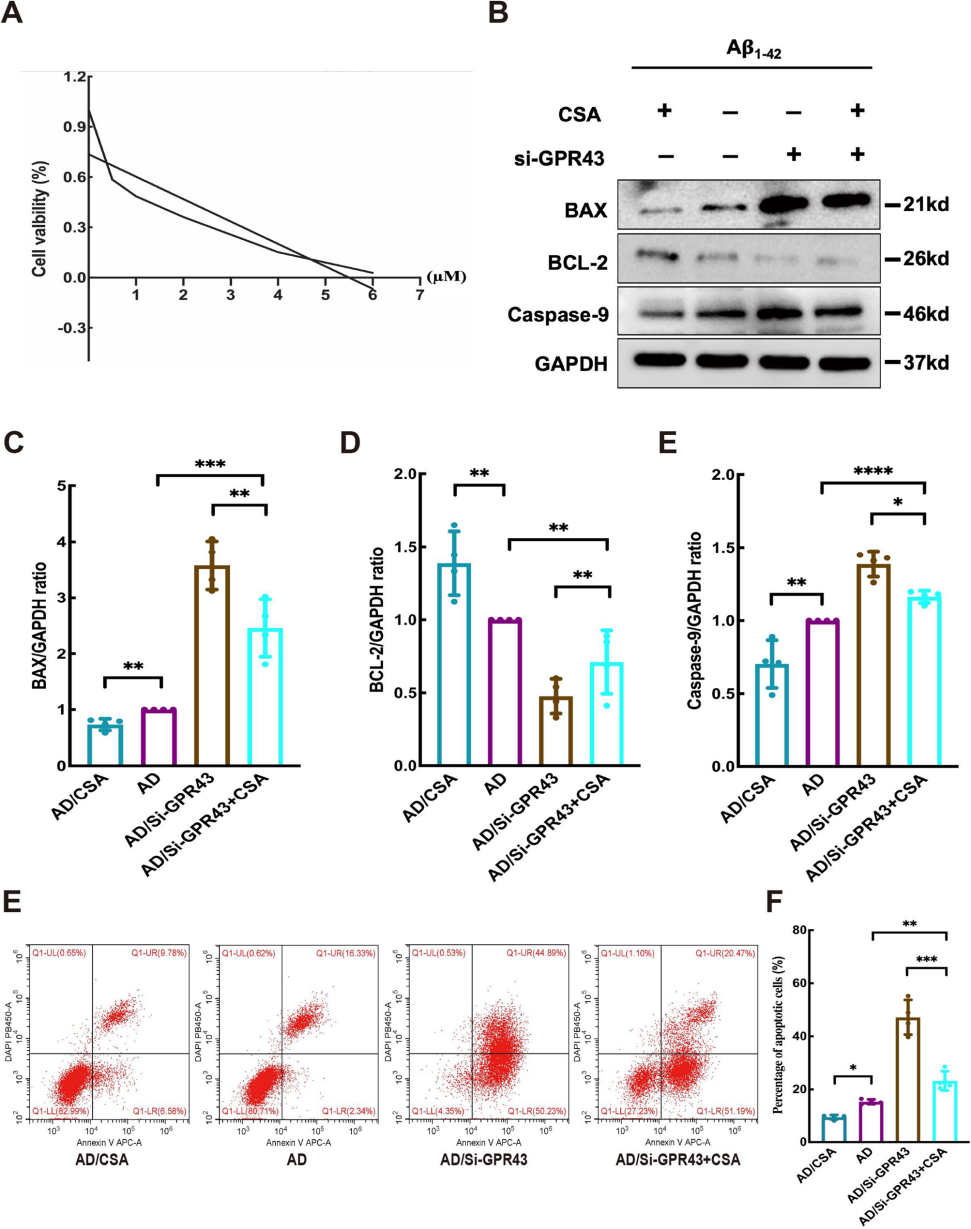
GPR43 modulates Alzheimer's disease (AD) pathology through the cyclophilin D (CypD) pathway. **A** Cell Counting Kit-8 **(**CCK-8) assay for determining optimal Cyclosporin A (CSA) concentration in HT22 cells. **B** Western blot analysis of apoptosis-related proteins. Quantification of (**C**) BCL2-Associated X (BAX), (**D**) B-cell lymphoma/leukemia 2 (BCL-2), and (**E**) Caspase-9 proteins levels. **F** Representative flow cytometry plots of Si-GPR43 cells treated with CSA. **G** Quantitative analysis of apoptosis rates. Data were presented as the mean ± SEM (*n* = 4). *p < 0.05, **p < 0.01, ***p < 0.001, and ****p < 0.0001

The CCK-8 viability assay identified 1 µM CSA as the optimal concentration for supporting cellular growth (Fig. 8A). Subsequence experiments treated Si-GPR43-transfected cells with this concentration alongside 10 µM Aβ_1–42_ for 48 h. Apoptotic protein profiling demonstrated that CSA treatment significantly modulated key regulators of cell death in the AD/Si-GPR43 model, with the AD/Si-GPR43 + CSA group showing reduced BAX levels(2.46 ± 0.26 vs. AD/Si-GPR43: 3.58 ± 0.22; *p* < 0.05; Fig. 8C), while exhibiting increased BCL-2 expression (0.71 ± 0.11 vs. 0.48 ± 0.06; *p* < 0.05; Fig. 8D) and decreased Caspase-9 expression (1.16 ± 0.02 vs. AD/Si-GPR43: 1.39 ± 0.04; *p* < 0.05; Fig. 8E). Flow cytometric analysis confirmed these findings, revealing a 49% reduction in apoptotic rates following CSA treatment (23.21 ± 1.76% in AD/Si-GPR43 + CSA vs. 47.23 ± 3.29 in AD/Si-GPR43; *p* < 0.05; Fig. 8G). These data collectively establish that pharmacological inhibition of CypD by CSA can effectively counteract the pro-apoptotic effects induced by GPR43 knockdown in Aβ_1–42_-treated neuronal cells.

## Discussion

This study reveals a novel neuroprotective role of GPR43 in AD pathogenesis. Our data demonstrate significant downregulation of GPR43 protein expression specifically in hippocampal and cortical neurons of Aβ_1−42_-induced AD model mice. Functional studies show that GPR43 activation modulates three key AD-related pathological features: (Apostolova 2016) neuronal viability (Selkoe 2011), mitochondrial function, and (Xu et al. 2022) synaptic functions. Mechanistically, we identified that GPR43 exerts neuroprotection by regulating mPTP dynamics via CypD-dependent signaling pathways, thereby attenuating neuronal apoptosis. These findings position GPR43 as a promising therapeutic target for AD intervention.

GPR43 likely plays a neuroprotective role in Aβ_1−42_-induced AD mouse models. Our experiments show that downregulation of GPR43 expression correlates with AD pathogenesis. While previous studies documented abundant GPR43 presence in various tissues, including the brain, our findings indicate its downregulation in the brains of AD, likely due to the disease. Furthermore, activating GPR43 via lentiviral injected into the brain significantly enhances memory and learning abilities, suggesting a protective effect of GPR43 in Aβ_1−42_-induced AD and proposing a novel hypothesis regarding its role in the disease.

GPR43 alleviates concurrent neuronal, mitochondrial, and synaptic dysfunction in AD mice, a crucial aspect of AD pathophysiology (Bindels et al. 2013; Qin et al. 2022; Mitchell et al. 2013; Finkel and Holbrook 2000; Verdin et al. 2010; Nakamura and Lipton 2010; Lee et al. 2004; Koffie et al. 2011). Our findings demonstrate that GPR43 overexpression enhances the secretion of BDNF, PSD95, and SYP compared to the AD group, confirming its role in neuronal and synaptic function. Histological analysis, including HE and Nissl staining, reveal that GPR43 overexpression reduces neuronal mortality. Immunofluorescence and Golgi staining indicate that GPR43 activation increases the density of neuronal synaptic axons in AD. Furthermore, electron microscopy shows that elevating GPR43 levels mitigates mitochondrial swelling and significantly alters LDH, MDA and SOD levels in AD. Collectively, these structural and functional changes suggest that neurons, mitochondria, and synapses interact to drive the pathophysiological alterations in the AD brain. We speculate that the deficiency of GPR43 in AD leads to imbalances in energy and calcium homeostasis, resulting in reduced mitochondrial energy production, diminished energy supply to neurons, ultimately compromising neuronal and synaptic function.

GPR43 colocalizes with neurons, but its localization in astrocytes or microglia is unclear. As is well known, brain cells mainly include neurons and glial cells. As a cell membrane protein, GPR43 can localize to the membrane of various cell types, fulfilling different roles based on disease pathology (Palczewski 2010; Bindels et al. 2013; Kimura et al. 2014). Accumulating evidence studies has demonstrated GPR43 localization to neuronal membranes, a finding concordant with our observation of GPR43-neurons co-staining in mice brain tissues (Fig. 1H). Notably, according to WU et al. reported, the mouse primary astrocytes have collocated withe GPR43 (Wu et al. 2024), contrasting with our data (Fig. 1H). However, the colocalization of GPR43 with microglia remains unclear. These discrepancies may arise from (i) interspecies variation in receptor expression profiles (e.g., murine vs. human models) or (ii) technical divergence in detection approaches (particularly antibody validation protocols).

GPR43 plays a complex role regulating apoptosis, which encompasses both endogenous and exogenous pathways (Razazan et al. 2021; Fang et al. 2019; Forman and Zhang 2021). The endogenous pathway involves mitochondria, caspase, and the BCL-2 family, while the exogenous pathway is influenced by Aβ deposition-activated microglia, that release inflammatory cytokines such as TNF-α, IL-6, and IL-1β (Naaldijk et al. 2017; Han et al. 2018). Recent studies have shown that GPR43 can alleviate apoptosis in lung cell (Forman and Zhang 2021; Xu et al. 2019) and inhibit apoptosis in myocardial cells (Yu et al. 2021). While these findings indicate a specific relationship between GPR43 and apoptosis, its role in AD remains unclear. To investigate whether GPR43 affects apoptosis in AD and the pathways involved, we examine apoptosis-related proteins such as Caspase-9, BAX, and BCL-2. Our results indicate that GPR43 overexpression effectively reverses levels of these proteins. Notably, TUNEL assays and flow cytometry reveal that GPR43 overexpression reduces the number of apoptotic bodies, suggesting that GPR43 overexpression reduces the number of apoptotic bodies, suggesting that GPR43 inhibits apoptosis in AD mice. Furthermore, our findings indicate that mitochondria, caspase-9, and BCL-2 family proteins are key triggers of the endogenous apoptosis pathways. Thus, we conclude that GPR43 regulates apoptosis primarily through endogenous pathways; however, the involvement of exogenous pathways remains to be determined. This discovery enhances our understanding of GPR43’s function, addressing a current gap in the literature.

GPR43 regulates mitochondrial function in AD mice by modulating mPTP dynamics. CypD, a crucial component for mPTP opening, was found to be elevated in AD mice but significantly inhibited in those overexpressing GPR43 compared to normal mice (Halestrap and Brenner 2003; Samanta et al. 2024). Our results indicate that GPR43 activation leads to suppressed CypD signaling, although the underlying mechanism remains unclear. Multiple studies suggest that GPR43’s downstream signaling pathways involve the activation of Gαi/o (cAMP-inhibitory) and Gαq (PLCβ/PKC-activating), increased intracellular calcium, and a reduction in pro-apoptotic signaling associated with mitochondrial stress (Saikachain et al. 2023; Kimura et al. 2020). However, the precise mechanism linking GPR43 and CypD requires further investigation. It is clear, though, that GPR43’s regulation of mitochondrial function is closely related to mPTP, presenting a novel perspective from this study.

CypD is a pivotal in mitochondrial-mediated apoptosis and has been linked to various neurological disorders, including AD, depression, epilepsy, and PD (Caspersen et al. 2005; Hauptmann et al. 2009; Bauer and Murphy 2020). In our study, AD mice overexpressing GPR43 exhibit reduced CypD protein levels and corresponding inhibition of apoptosis compared to AD mice, indicating a close relationship between CypD and apoptosis. This association likely stems from the role of CypD as a key component of mitochondrial mPTP, where it acts as a regulatory factor that facilitates mPTP opening by binding to ATP5B. The opening of mPTP results in osmotic swelling, dissipation of mitochondrial membrane potential (∆ Ψ m), decreased mitochondrial calcium retention capacity, and increased production of reactive oxygen species (ROS), ultimately promoting cell apoptosis and death. Therefore, we conclude that CypD plays a critical role in mitochondrial-mediated apoptosis.

GPR43 regulates apoptotic processes in AD through modulation of the CypD signaling pathway. To experimentally validate this mechanism, we employed CSA, a specific CypD inhibitor, to determine whether pharmacological blockade of CypD could rescue the pro-apoptotic effects induced by GPR43 knockdown in an AD model system. Our data indicate that CSA significantly reduces the apoptosis rate, lowers BAX and caspase-9 levels, and increases BCL-2 levels associated with GPR43 knockdown. Overall, CSA effectively alleviates apoptosis in cells with GPR43 knockdown in Aβ_1−42_ environments. These results validate that GPR43 influences AD pathogenesis through the CypD signaling pathway, though the specific mechanisms warrant further investigation.

GPR43 presents a promising target for AD treatment. Previous studies indicate that apoptotic pathways contribute to the disease’s onset and progression, highlighting their potential as therapeutic targets for AD (Marzo et al. 1998; Calissano et al. 2009). Our study demonstrates that decreased GPR43 levels elevate CypD, triggering mPTP opening. This cascade leads to mitochondrial dysfunction, neuronal cell death, synaptic damage, and impaired memory and learning abilities in mice, thereby promoting apoptosis in AD pathology. Conversely, lentiviral-mediated upregulation of GPR43 suppresses CypD activation, ultimately reducing mitochondrial injury, neuronal loss, synaptic damage, and apoptosis. These insights into GPR43’s role in apoptosis enhance our understanding of the mechanisms of AD pathology and reveal that GPR43 acts as a sensor for CypD-mediated mitochondrial apoptosis, which may offer novel perspectives for AD treatment strategies.

**Fig. 9:**
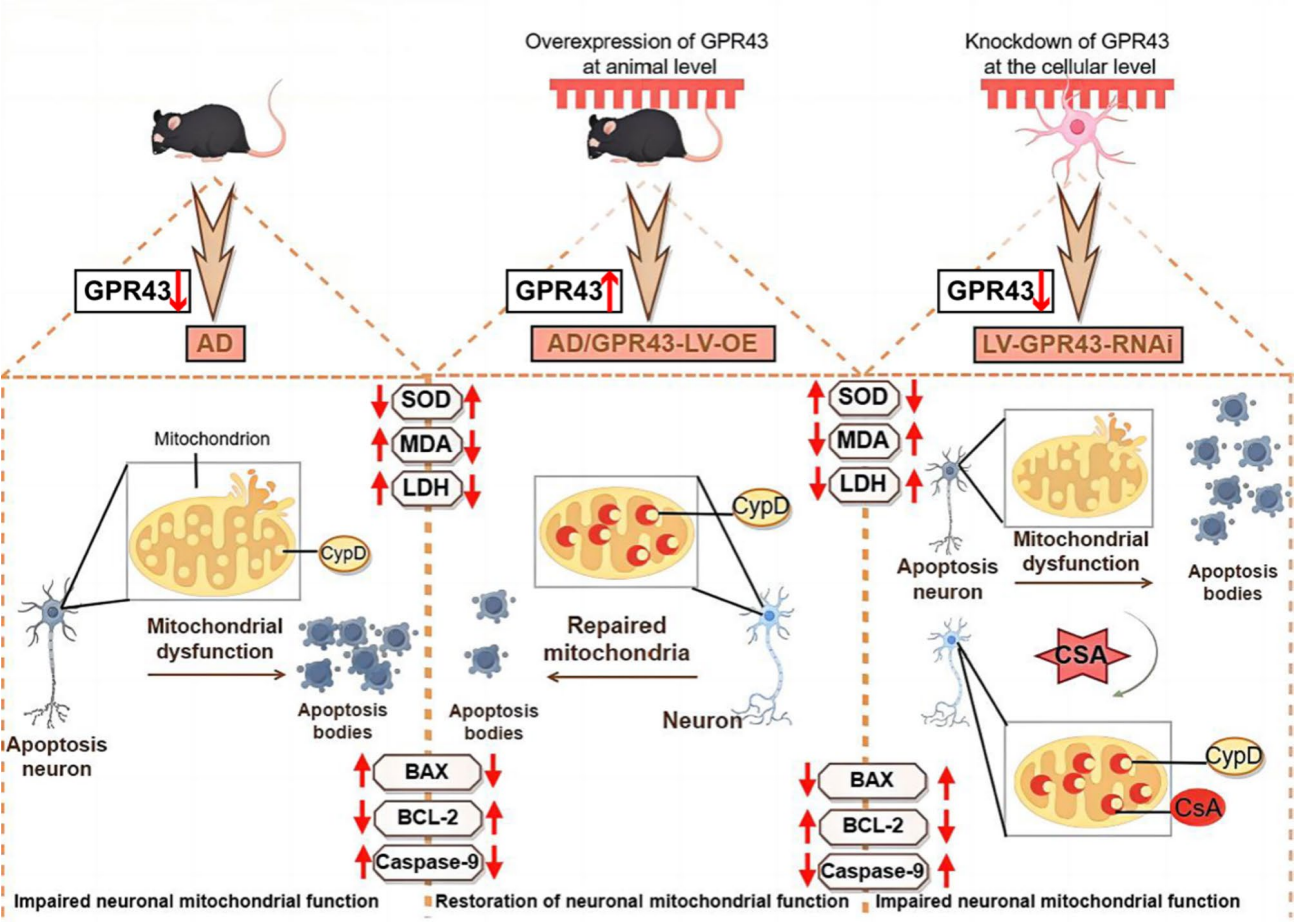
GPR43-CypD axis controls mitochondrial apoptosis in Alzheimer’s disease (AD) pathogenesis. In AD mice with GPR43 overexpression (GPR43-LV-OE), mitochondrial damage was attenuated, and neuronal apoptosis was reduced. Conversely, GPR43 knockdown exacerbated mitochondrial dysfunction and promoted neuronal apoptosis. Treatment with Cyclosporin A (CSA), a CypD inhibitor, restored mitochondrial function and suppressed the neuronal apoptotic pathway

In conclusion, our study provides new evidence that GPR43 can improve mitochondrial function, reduce neuronal and synaptic damage, and inhibit apoptosis by inhibiting CypD activity, thereby improving cognitive and learning dysfunction in AD mice. These findings reveal the role of GPR43 in AD and present new therapeutic avenues. The schematic mechanism is shown in Fig. 9.

## Data Availability

No datasets were generated or analysed during the current study.
